# Impact of a Structured Faculty Development Program on Reducing Examiner Variability in Undergraduate Medical Theory Assessments: A Quasi-Experimental Study

**DOI:** 10.7759/cureus.105946

**Published:** 2026-03-26

**Authors:** Sukanti Bhattacharyya, Alka Rawekar, Arkaprabha Sau, Damodar P Goswami, S Samashaptak

**Affiliations:** 1 Department of Medical Physiology, ICARE Institute of Medical Sciences and Research and Dr. Bidhan Chandra Roy Hospital, Haldia, IND; 2 Department of Physiology, Jawaharlal Nehru Medical College, Datta Meghe Institute of Higher Education and Research (Deemed to be University), Wardha, IND; 3 Department of Industrial Medicine, Regional Labour Institute, Kolkata, IND; 4 Department of Research, Sivatosh Mookerjee Science Centre, Asutosh Mookerjee Memorial Institute, Kolkata, IND; 5 Department of General Medicine, IraSunil Clinic, Kolkata, IND

**Keywords:** development, education, faculty, medical, program

## Abstract

Introduction: Examiner variability in medical theory assessments might compromise fairness, reliability, and learner trust. The present study aimed to evaluate the effect of a structured faculty development program (FDP) on reducing examiner variability and improving scoring consistency in undergraduate medical theory assessments using short essay questions (SEQs) and multiple-choice questions (MCQs).

Materials and methods: This quasi-experimental study involved 32 experienced physiology faculty members from medical colleges affiliated with a state health university in India. Participants were divided into a trained group (n = 16) and an untrained control group (n = 16). All examiners first scored identical anonymized answer scripts (one SEQ and ten MCQs) under standard conditions without rubrics or answer keys. Following a three-week washout period, the trained group participated in a structured faculty development program focused on assessment literacy, rubric design, examiner calibration, and reflective practices. Both groups then re-scored the same scripts. Intra-rater consistency, inter-rater reliability (intraclass correlation coefficients as ICC and Fleiss’ kappa), and scoring accuracy were compared pre- and post-intervention.

Results: The structured FDP significantly reduced examiner variability in undergraduate medical theory assessments. Trained examiners showed marked improvements in intra-rater consistency, inter-rater reliability (SEQ ICC from 0.79 to 0.91; MCQ Fleiss’ kappa from 0.16 to 0.33), and MCQ accuracy (48% to 88%). Variability decreased substantially in the trained group, while the untrained group exhibited minimal change or slight decline. Significant group × time interaction (p = 0.013 for SEQs) and group main effect (p = 0.043 for MCQs) confirmed the intervention’s effectiveness. These findings highlight the value of targeted FDPs in enhancing assessment fairness and reliability in medical education.

Conclusion: A targeted, short-duration FDP effectively reduced examiner variability and enhanced reliability in undergraduate theory assessments. Implementing such programs can promote fairer, more defensible evaluations in medical education, particularly in resource-constrained settings.

## Introduction

Examiner variability remains a persistent challenge in medical education, particularly in theory assessments where subjective judgment plays a significant role. This variability arises primarily from differences in examiner stringency (the “hawk” tendency) and leniency (the “dove” tendency), collectively known as the Hawk-Dove effect [1]. Such inconsistencies can lead to unfair grading, reduced assessment reliability, and diminished learner trust. In high-stakes medical training, where examination outcomes influence progression, licensure, and ultimately patient safety, examiner-related bias undermines the principles of fairness, transparency, and defensibility [2].

Research has consistently demonstrated that examiner variability persists across various assessment formats, including structured clinical examinations, performance-based assessments, and written theory papers [3]. Factors contributing to these variabilities include cognitive biases, mood, prior expectations, and individual interpretive styles [4,5]. Faculty development programs (FDPs) focused on assessment literacy, rubric design, and examiner calibration have been proposed as effective interventions to enhance consistency [6]. While evidence supports their role in clinical and postgraduate settings, data on their impact in undergraduate medical theory examinations, particularly involving short essay questions (SEQs) and multiple-choice questions (MCQs), remain limited. This gap is critical, as theory assessments form a major component of undergraduate evaluation, and variability in these formats can disproportionately affect student performance. The aim of this study was to evaluate the effect of a structured FDP on reducing examiner variability in undergraduate medical theory assessments using SEQs and MCQs. The primary objective was to determine the impact of the FDP on intra-rater consistency, inter-rater reliability, and overall scoring behavior. The secondary objective was to examine variability in MCQ scoring in the absence of standardized answer keys and to identify patterns of examiner inconsistency.

## Materials and methods

This study employed a non-randomized, pre-post interventional (quasi-experimental) design. The design allowed comparison of examiner scoring behavior before and after the intervention within the trained group, while using an untrained group as a concurrent comparison to assess baseline inter-rater differences. This approach was chosen for its practicality in a real-world academic setting, where random allocation of experienced examiners to training conditions could disrupt ongoing examination responsibilities.

Study setting

The study was conducted from April 2025 to June 2025 in the physiology departments of various medical colleges affiliated with the West Bengal University of Health Sciences (WBUHS), India. These departments follow a uniform undergraduate MBBS curriculum and conduct regular theory examinations as per university guidelines. Data collection occurred during routine academic assessment periods to preserve ecological validity and ensure that examiner behavior reflected authentic marking conditions. No alterations were made to institutional examination schedules or examiner duties.

Sample size estimation

A priori sample size estimation was performed using G*Power 3.1 software (Heinrich-Heine-Universität Düsseldorf in Düsseldorf, Germany). A point-biserial correlation test was selected to detect a moderate effect size (r = 0.40). With an alpha level set at 0.05 (two-tailed) and a desired power of 80%, the analysis indicated a required sample size of 32 examiners. This calculation accounted for the correlation between pre- and post-intervention scores within subjects.

Participants

Thirty-two senior physiology faculty members with at least 10 years of undergraduate teaching and assessment experience served as examiners. All were regularly involved in theory paper evaluation. Participation was voluntary, with no incentives provided. The use of experienced examiners ensured that observed scoring patterns reflected mature judgment rather than novice variability. After baseline scoring, examiners were non-randomly assigned: 16 to the intervention (trained) group and 16 to the control (untrained) group.

Assessment materials

Anonymized answer scripts from ten Bachelor of Medicine and Bachelor of Surgery (MBBS) students were used solely to study examiner behavior; the students were not study participants, and their responses did not influence academic progression or summative evaluation. The assessment comprised one short essay question (SEQ) and ten multiple-choice questions (MCQs) on the gastrointestinal system, a core topic in undergraduate physiology. Questions were randomly selected from must-know areas and were originally framed by a senior physiology professor with over 25 years of experience. At baseline, no standardized rubrics or MCQ answer keys were provided.

Intervention

All 32 examiners initially scored the same set of anonymized answer scripts under standard (untrained) conditions, without rubrics or answer keys, to establish baseline scoring data. Following a three-week washout period designed to minimize carryover and recall effects, 16 examiners participated in a structured FDP focused on assessment reliability, SEQ rubric development and application, examiner calibration exercises, and reflective assessment practices.

The FDP was conducted as a structured, six-hour, single-day workshop delivered in four sequential modules. The first module (1.5 hours) focused on assessment literacy and reliability, covering principles of validity, inter-rater and intra-rater reliability, the hawk-dove effect, and common sources of examiner bias in written theory assessments. The second module (1.5 hours) addressed analytic rubric development for SEQs, including blueprinting, domain weight allocation, model answer preparation, and structured marking schemes. The third module (2 hours) consisted of examiner calibration exercises, during which participants independently scored anonymized sample scripts (different from study scripts), followed by facilitated group discussions to identify scoring discrepancies and achieve consensus on marking standards. The final module (1 hour) focused on MCQ standardization and reflective practice, emphasizing validated answer key development, identification of ambiguous stems and distractors, and recognition of cognitive biases influencing judgment. The FDP employed interactive teaching strategies, including small-group work, guided rubric drafting, scoring comparison exercises, and structured consensus-building discussions. Participants were provided with sample rubrics, model answer keys, and reliability interpretation guides.

The program was designed to enhance judgment consistency without intentionally altering grading severity. After the intervention, all 32 examiners (both trained and untrained groups) re-scored the identical anonymized scripts under the same standard conditions as at baseline. To reduce recall bias, examiners were not informed of their previous scores. The untrained group received no intervention and served as the concurrent control for inter-rater comparisons. Scoring was performed independently in controlled conditions mimicking real examination marking. Scripts remained fully anonymized throughout. All scoring took place within the examiners' routine academic workload.

Statistical analysis

Data were analyzed using standard statistical software, IBM Corp. Released 2017. IBM SPSS Statistics for Windows, Version 23. Armonk, NY: IBM Corp. Descriptive statistics summarized examiner scores (means, standard deviations). Paired t-tests assessed intra-rater changes in the trained group (pre- vs. post-FDP mean scores). Mixed analysis of variance (ANOVA) examined variability between examiners for section-wise (SEQs and MCQs). Inter-rater reliability was quantified using intraclass correlation coefficients (ICC), specifically ICC for single measures and ICC for average measures. MCQ agreement was expressed as a percentage concordance across examiners. A p-value < 0.05 was considered statistically significant.

Ethical considerations

The study adhered to the Declaration of Helsinki and institutional ethical standards. Approval was obtained from the Institutional Ethics Committee of ICARE Institute of Medical Sciences and Research, Haldia, West Bengal (IIMSAR-Haldia/IEC/March 2025/01). Informed consent was secured from all participating examiners. Student scripts were anonymized and used only as assessment stimuli; students were not considered participants, and no identifiable data were collected or used. Confidentiality of both examiners and scripts was maintained throughout.

## Results

Baseline characteristics of 16 (50%) trained and 16 (50%) untrained (total 32 examiners) were analyzed using independent t-tests. Both groups demonstrated comparable experience levels (trained: 14.0 ± 1.8 years; untrained: 14.0 ± 1.4 years; p = 0.876). No statistically significant differences existed in baseline assessment performance: SEQ scores (p = 0.310), MCQ scores (p = 0.486), or deviation counts (p = 0.762). These findings confirm effective baseline group equivalence, indicating that any post-intervention differences can be attributed to the FDP training rather than pre-existing examiner characteristics or initial scoring ability disparities (Table 1).

**Table 1:** Baseline characteristics and assessment scores of examiners in trained and untrained groups. Values are presented as mean ± standard deviation (SD). Baseline comparability between groups was assessed using independent samples t-tests, SEQ: short essay question (minimum marks 0, maximum marks 10), and MCQ: multiple-choice question (minimum marks 0, maximum marks 1). A p-value > 0.05 was considered non-statistically significant. Deviation count: how many times an examiner's rating/score differed from a correct answer?

Characteristic	Trained Group (n = 16)	Untrained Group (n = 16)	Total (n = 32)	T stats	p-value
Experience in years (Mean ± SD)	14.00 ± 1.80	14.00 ± 1.40	14.00 ± 1.50	0.06	0.876
Baseline SEQ score (Mean ± SD)	5.75 ± 0.96	6.00 ± 0.82	5.88 ± 0.84	0.44	0.310
Baseline MCQ score (Mean ± SD)	5.00 ± 0.41	5.75 ± 1.26	5.38 ± 1.25	0.29	0.486
Baseline deviation count for MCQs (Mean ± SD)	3.75 ± 1.26	3.50 ± 0.58	3.63 ± 0.92	0.40	0.762

The structured FDP demonstrated significant positive effects on assessment accuracy. Trained examiners showed statistically significant improvements in both SEQ scoring (mean difference: +1.25, p = 0.049) and MCQ accuracy (mean difference: +0.50, p = 0.043) following the intervention. In contrast, untrained examiners exhibited non-significant declines in both metrics (-0.50 SEQ, -0.25 MCQ; p > 0.05). These findings indicate that the FDP effectively enhanced scoring consistency and reduced variability among trained faculty. The divergent trajectories between groups suggest that observed improvements can be attributed to the training intervention rather than practice effects or time-related factors alone, supporting the FDP's role in standardizing assessment practices (Table 2).

**Table 2:** Pre- and post-intervention SEQ and MCQ scores among trained and untrained examiners. Values are presented as mean (SD). Within-group pre- and post-intervention comparisons were performed using paired t-tests. Positive mean differences indicate improvement following the intervention. *p < 0.05 denotes statistical significance. SEQ: short essay question, MCQ: multiple-choice question, FDP: faculty development program.

Parameters	Groups	Pre-intervention mean (SD)	Post-intervention mean (SD)	Mean Difference (Post - Pre)	T stats	p-value
SEQ	Trained (n=16)	5.75 ± 0.96	7.00 ± 0.82	+1.25	2.21	0.049*
	Untrained (n=16)	6.00 ± 0.82	5.50 ± 0.58	-0.50	1.11	0.291
MCQ	Trained (n=16)	5.00 ± 1.41	6.25 ± 1.09	+0.50	0.65	0.043*
	Untrained (n=16)	5.75 ± 1.26	5.50 ± 1.66	-0.25	0.78	0.482

**Figure 1:**
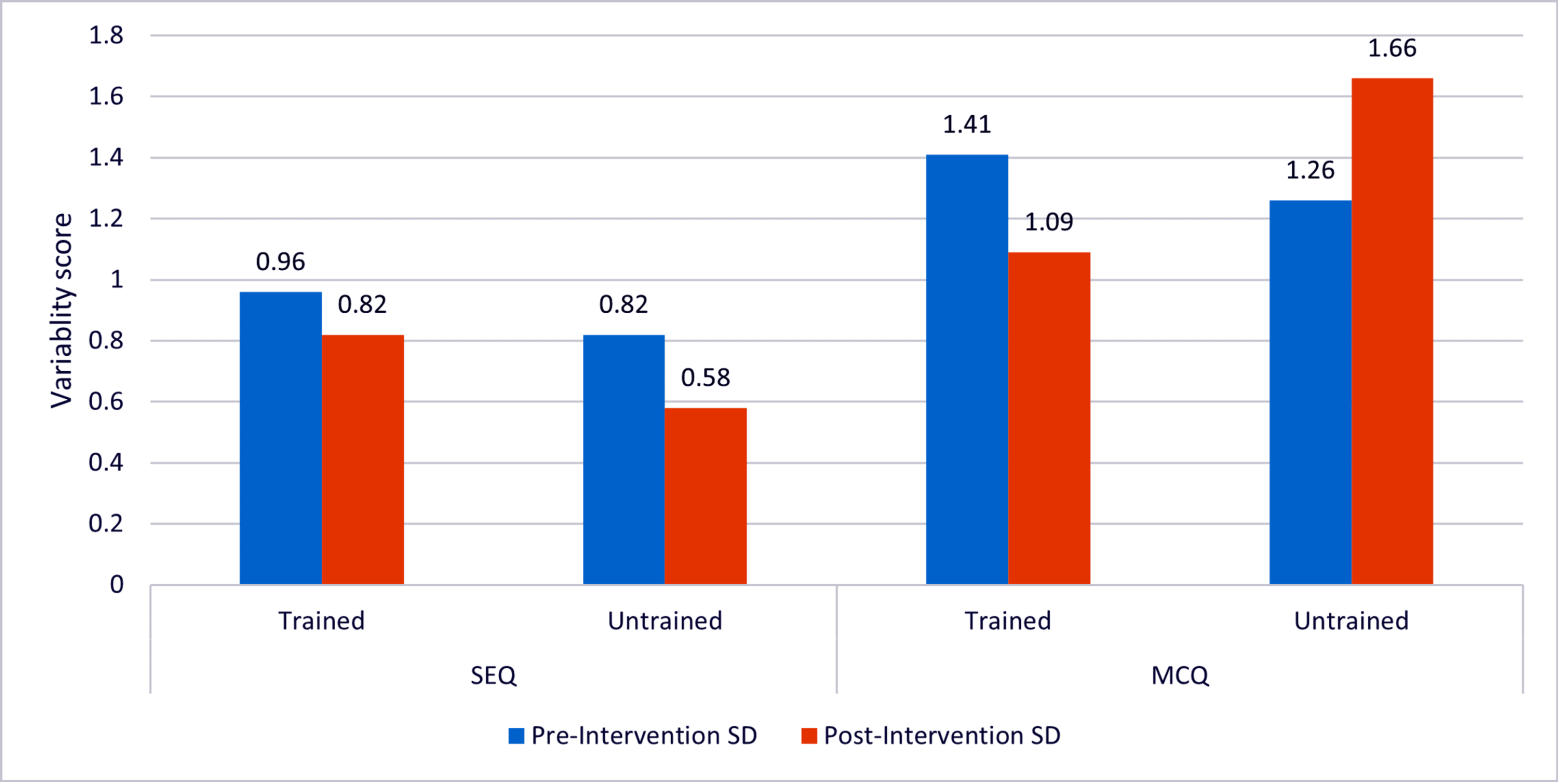
Changes in inter-rater variability of short essay question (SEQ) and multiple-choice question (MCQ) scores before and after the faculty development program (FDP). SD: Standard deviation.

Post-intervention variability decreased markedly for trained examiners, with MCQ standard deviation reducing from 1.41 to 1.09, while untrained examiners showed an increase in variability from 1.26 to 1.66. For SEQs, both groups demonstrated modest improvement in consistency. These results indicate that structured FDP training significantly reduced inter-rater variability, particularly in objective MCQ scoring. The pronounced reduction among trained examiners highlights the effectiveness of standardized answer keys and calibration exercises in promoting uniform assessment practices, whereas the persistence of variability in the untrained group underscores the necessity of formal training to achieve scoring consistency (Figure 1).

The mixed ANOVA revealed a significant group × time interaction for SEQ scores (p = 0.013), indicating that the FDP training differentially influenced scoring patterns between groups. For MCQ scores, a significant main effect of group was observed (p = 0.043), demonstrating that trained examiners maintained higher overall accuracy. The absence of significant main effects for time in both measures suggests that simple retesting alone did not improve performance. These results collectively indicate that the structured FDP specifically enhanced inter-rater consistency and scoring behavior, with distinct impacts on different assessment formats, improving standardized marking in MCQs while modifying scoring approaches in SEQs (Table 3).

**Table 3:** Mixed analysis of variance (ANOVA) examining the effects of faculty development training and time on SEQ and MCQ scores. Mixed-design ANOVA assessed the main effects of Group (trained vs. untrained), Time (pre- vs. post-intervention), and their interaction (Group × Time), SEQ: short essay question, MCQ: multiple-choice question, *p < 0.05 indicates statistical significance, df: degree of freedom.

Outcome measure	Source of Variance	Sum of squares	df	Mean square	F stats	p-value
SEQ scores	Group	0.06	1	0.06	0.05	0.836
	Time	1.56	1	1.56	3.38	0.115
	Group × Time	0.56	1	0.56	1.22	0.013*
MCQ total scores	Group	0.06	1	0.06	1.03	0.043*
	Time	0.56	1	0.56	1	0.356
	Group × Time	0.06	1	0.06	0.11	0.751

The FDP intervention significantly enhanced inter-rater reliability metrics for trained examiners. Post-training, the trained group's Fleiss' Kappa for MCQs improved from 0.16 (slight agreement) to 0.33 (fair agreement), while their SEQ intraclass correlation coefficient increased from 0.79 to 0.91. These findings demonstrate that structured examiner training effectively improved scoring consistency and agreement among trained faculty, while the absence of such training led to deteriorating inter-rater reliability, highlighting the FDP's crucial role in standardizing assessment practices and reducing subjective variability in medical education evaluations (Table 4).

**Table 4:** Inter-rater agreement indices for SEQ and MCQ scoring before and after the intervention. Inter-rater agreement for MCQs was assessed using Fleiss’ Kappa, interpreted according to standard benchmarks. SEQ reliability was evaluated using intraclass correlation coefficients (ICC) with 95% confidence intervals (CI). Positive change values indicate improvement in agreement following the intervention. SEQ: short essay question, MCQ: multiple-choice question. Change scores (post - pre) represent within-subject differences; therefore, ICCs for change are typically derived from difference score parameters. Confidence intervals for change score ICCs could not be calculated due to negative variance components and the nature of difference score distributions.

Time	Group	Fleiss' Kappa for MCQ	Interpretation	SEQ ICC (95% CI)
Pre	Trained	0.16	Slight agreement	0.79 (0.387-0.964)
	Untrained	0.20	Slight agreement	0.83 (0.480-0.971)
Post	Trained	0.33	Fair agreement	0.91 (0.705-0.989)
	Untrained	0.13	Slight agreement	0.75 (0.304-0.959)
Change	Trained	0.16	Improved	0.12
	Untrained	-0.07	Worsened	-0.08

Pre-intervention analysis revealed substantial variability in MCQ scoring among examiners. Items MCQ4 and MCQ5 showed the highest inconsistency with even 50/50 splits (variance = 0.25), indicating complete examiner disagreement. Most items demonstrated moderate inconsistency (6/10 items), while only MCQ2 and MCQ8 exhibited relatively low variability. The direction of bias varied, with some items skewed toward the correct answer and others away from it. These findings highlight significant baseline examiner disagreement in the absence of standardized answer keys, underscoring the inherent subjectivity in MCQ evaluation and emphasizing the critical need for structured training and validated scoring keys to ensure assessment reliability (Table 5).

**Table 5:** Item-wise variability in MCQ scoring prior to intervention among all examiners. Mean scores represent the proportion of examiners awarding the intended correct response (range 0–1). Binomial variance reflects examiner disagreement for each item, with higher values indicating greater inconsistency. Direction of bias refers to examiner tendency toward or away from the intended correct answer. MCQ: multiple-choice question.

MCQ	Mean score	Binomial variance	Interpretation of inconsistency
MCQ1	0.63	0.23	Moderate; biased toward correct
MCQ2	0.75	0.19	Low; biased opposite correct
MCQ3	0.63	0.23	Moderate; biased toward correct
MCQ4	0.50	0.25	High; even split
MCQ5	0.50	0.25	High; even split
MCQ6	0.63	0.23	Moderate; biased opposite correct
MCQ7	0.38	0.23	Moderate; biased opposite correct
MCQ8	0.75	0.19	Low; biased toward correct
MCQ9	0.63	0.23	Moderate; biased toward correct
MCQ10	0.63	0.23	Moderate; biased opposite correct

**Figure 2:**
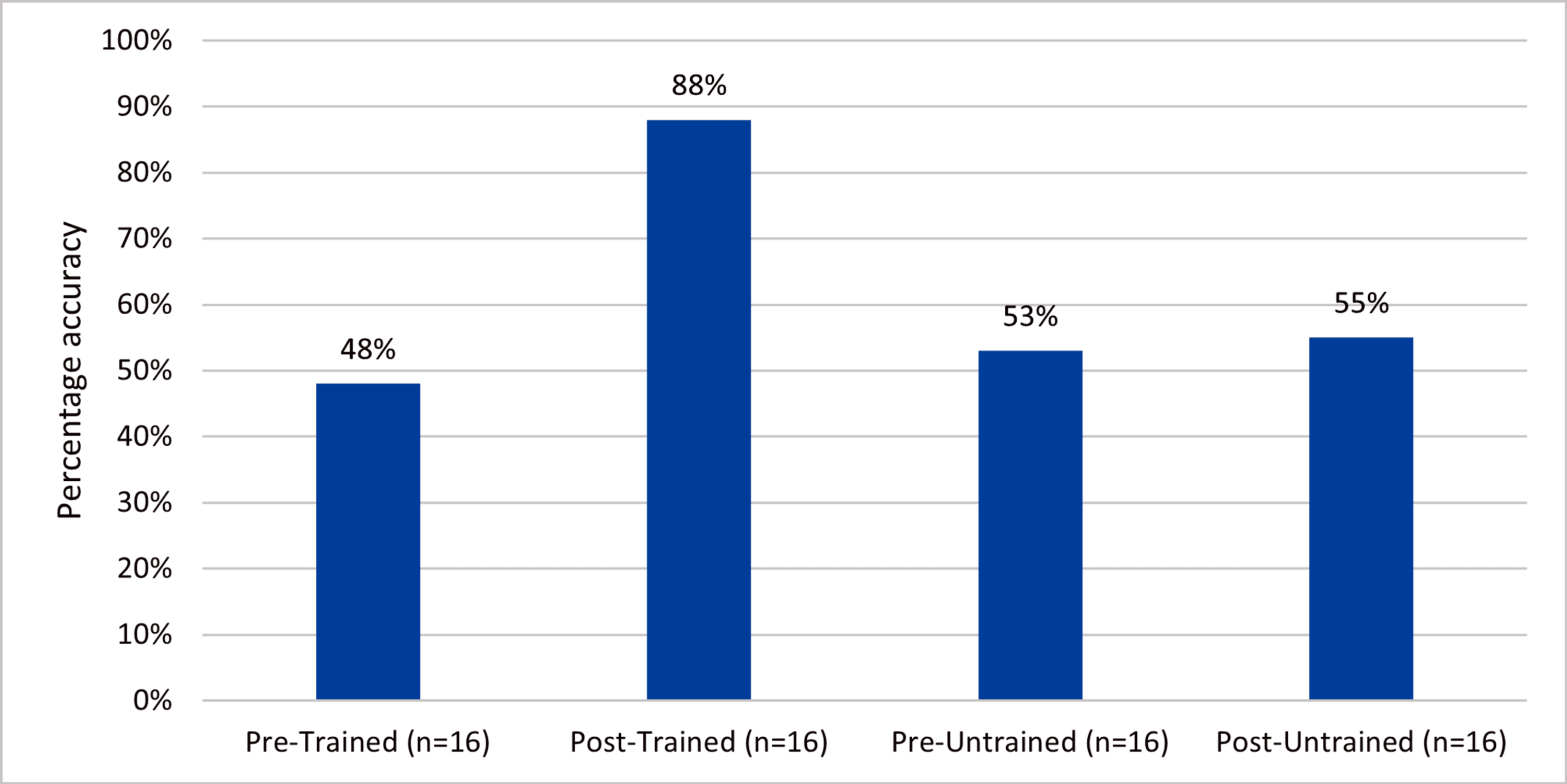
Examiner accuracy in MCQ scoring relative to intended correct answers before and after the intervention. MCQ: Multiple choice questions

The structured FDP produced a dramatic improvement in MCQ scoring accuracy. Trained examiners increased from 48% to 88% accuracy post-intervention, representing a substantial 40-percentage-point gain and near-perfect alignment with intended answers. In contrast, untrained examiners showed minimal change (53% to 55%), confirming that the improvement was not attributable to practice or time effects alone. This 33-percentage-point differential between groups provides compelling evidence that the training program effectively calibrated examiner judgment, significantly reducing errors and enhancing assessment fidelity when standardized answer keys were absent (Figure 2).

## Discussion

The findings of this study underscore the persistent challenge of examiner variability in undergraduate medical theory assessments and highlight the potential of structured FDPs to mitigate such inconsistencies. The observed improvements in scoring accuracy and consistency among trained examiners suggest that targeted interventions can effectively address subjective biases, aligning examiner judgments more closely with standardized practices. This is particularly evident in the enhanced alignment for MCQs, where the absence of predefined keys initially amplified discrepancies, and in SEQs, where rubric-based calibration refined evaluative approaches.

These outcomes resonate with existing literature on rater training in medical education. For instance, a study by Yeates and Sebok-Syer [3] demonstrated that examiner calibration workshops (Many Facet Rasch Modeling) reduced hawk-dove effects in objective structured clinical examinations (OSCEs), though the effect was small, attributing improvements to heightened awareness of cognitive biases and standardized scoring frameworks. Similarly, Owolabi et al. [7] reported enhanced inter-rater reliability in written assessments following faculty training, with ICCs improving from moderate to excellent levels, mirroring the shifts seen here. Similar results were reported in another study where significant improvement in participants' MCQ writing skills was noted after FDP [8].

The differential impact on MCQs versus SEQs aligns with research by Downing [9], who noted that objective formats such as MCQs are more susceptible to interpretive variability without keys, while subjective SEQs benefit from rubric training to curb leniency or stringency. Gupta et al. [10] reported that a single faculty training program did not bring any significant improvement in the writing of MCQs, and they advised conducting multiple FDPs to combat the inconsistencies.

The mechanisms underlying these improvements likely involve multiple facets of the intervention. By incorporating assessment literacy and calibration exercises, the FDP fostered a shared understanding of evaluation criteria, reducing individual interpretive styles that fuel the hawk-dove phenomenon. However, our findings were in contradiction with a previous study where rater training did not improve inter-rater reliability or accuracy of mini-clinical evaluation exercise scores [11]. Moreover, the washout period and anonymization protocols likely isolated the training effect, as evidenced by the lack of similar progress in the untrained group. Cook et al. [12] reported that simulation-based training led to a significant improvement in knowledge and behavior compared to the control group with no intervention.

Broader contextual factors also justify these results. In India, which hosts the highest number of medical colleges globally and consequently a large cadre of medical teachers, FDPs remain inconsistent and fragmented, with significant gaps in coverage, content, and institutional commitment [13]. This highlights the urgent need for structured, evidence-based interventions to enhance teaching and assessment competencies. The present study addresses this critical shortfall by demonstrating measurable gains in inter-rater reliability and scoring accuracy through a targeted, short-duration FDP in undergraduate physiology theory assessments. Steinert et al. [14] included 48 studies and found that faculty members were highly satisfied professionally and personally with FDPs. They reported increased knowledge of leadership concepts, principles, gains in specific leadership skills, and increased awareness of leadership roles in academic settings.

Christie et al. [15] reported that targeted faculty development significantly improved consistency and quality in clinical evaluation of student competence in ethical reasoning and professionalism, emphasizing that calibration and structured guidance reduce subjective variability among evaluators. Their findings reinforce the current results, suggesting that examiner training enhances judgment alignment and promotes more defensible assessment practices across educational contexts. These findings are further supported by Lombarts et al. [16], who demonstrated that a positive and structured learning climate within residency programs was significantly associated with improved faculty teaching performance as evaluated by residents. Their study highlights that faculty performance is not static but responsive to institutional and educational interventions that promote reflection, feedback, and professional development. This aligns with the present findings, suggesting that structured faculty development initiatives, including examiner calibration and assessment literacy training, can meaningfully enhance faculty performance in assessment contexts and contribute to more reliable and equitable evaluation practices.

The clinical implications of these findings are profound for medical education and patient care. By standardizing theory assessments, FDPs can enhance the fairness of high-stakes evaluations, ensuring that student progression reflects true competency rather than examiner bias. This, in turn, bolsters the defensibility of licensure processes, ultimately contributing to safer healthcare delivery as graduates enter practice with more reliable foundational knowledge. Institutions should integrate such programs into routine faculty development to promote equity, particularly in diverse curricula.

Limitations include the non-randomized allocation, which, while pragmatic, may introduce unmeasured confounders such as motivation differences between groups. The sample, drawn from physiology departments, limits generalizability to other specialties or regions. Additionally, the short-term follow-up precludes assessment of long-term retention of training effects, and reliance on anonymized scripts may not fully capture real-time marking dynamics. Future research could employ randomized trials across multiple subjects and incorporate longitudinal tracking to validate sustained impacts. Despite these constraints, this study provides compelling evidence for FDPs as a scalable solution to examiner variability.

## Conclusions

This study demonstrated that a structured FDP significantly reduced examiner variability and enhanced scoring consistency in undergraduate medical theory assessments involving SEQs and MCQs. Trained examiners showed substantial improvements in intra-rater reliability, inter-rater agreement, and MCQ accuracy, driven by rubric application and calibration exercises, while the untrained group exhibited persistent inconsistencies. These findings highlight the value of targeted training in addressing hawk-dove tendencies and subjective biases. In the Indian context, where faculty development remains inconsistent, implementing regular, focused FDPs offers a practical, evidence-based approach to promote assessment fairness, reliability, and equity. Ultimately, such interventions support more defensible evaluations and contribute to producing competent, fairly assessed medical graduates.
